# Proposal for a Method for Assessing the Quality of an Updated Deep Learning-Based Automatic Segmentation Program

**DOI:** 10.7759/cureus.81307

**Published:** 2025-03-27

**Authors:** Fumihiro Tomita, Ryohei Yamauchi, Shinobu Akiyama, Miki Hirano, Tomoyuki Masuda, Satoshi Ishikura

**Affiliations:** 1 Department of Radiation Oncology, St. Luke’s International Hospital, Tokyo, JPN

**Keywords:** auto-segmentation, deep learning (dl), radiation therapy contouring, radiation therapy (rt), roi

## Abstract

This study aimed to verify whether a commercial deep learning-based automatic segmentation (DLS) method can maintain contour geometric accuracy post-update and to propose a streamlined validation method that minimizes the burden on clinical workflows. This study included 109 participants. Radiation oncologists used computed tomography (CT) imaging to identify 28 organs located in the head and neck, chest, abdomen, and pelvic regions. Contours were delineated on CT images using AI-Rad Companion Organs RT (AIRC; Siemens Healthineers, Erlangen, Germany) versions VA30, VA50, and VA50. The Dice similarity coefficient, maximum Hausdorff distance, and mean distance to agreement were calculated to identify contours with significant differences among versions. To evaluate the identified contours, the ground truth was defined as the contour delineated by radiation oncologists, and the geometric indices for VA30, VA50, and VA60 were recalculated. Statistical analysis was performed on the geometric indices to verify differences between each version. Among the 28 contours evaluated, nine organs did not satisfy the established criteria. Statistical analysis revealed that the brain, rectum, and bladder contours differed substantially across AIRC versions. In particular, the pre-update rectum contour had a mean (range) Hausdorff distance of 0.76 (0.40-1.16), whereas the post-update rectum contour exhibited lower quality, with a Hausdorff distance of 1.13 (0.24-5.68). Therefore, commercial DLS methods that undergo regular updates must be reassessed for quality in each region of interest. The proposed method can help reduce the burden on clinical workflows while appropriately evaluating post-update DLS performance.

## Introduction

The contours of tumors and organs at risk are essential for radiotherapy planning. However, the manual delineation of contours is time-consuming, even for experienced radiation oncologists. Moreover, anatomical structures may change because of tumor responses or body weight variations, necessitating frequent updates. Although manual contour delineation remains crucial in clinical workflows, interobserver variability is a widely reported issue [1-3]. This subjective variation increases uncertainty in treatment planning by affecting dose distribution and treatment optimization [4].

In response to these concerns, automatic segmentation has emerged as a new contouring technology [5], particularly deep learning (DL)-based automatic segmentation (DLS) [6,7]. DL reveals hidden patterns and relationships by hierarchically extracting features from images and training them iteratively. Commercial DLS reduces the time needed for manual contour delineation by 8.5 min [8]. Additionally, DLS has been shown to reduce interobserver variability and shorten the mean time required for head and neck contour delineation by 16.2 min [9]. Several studies have reported the effectiveness of DLS in clinical practice.

However, DLS programs are limited by their difficulty in generalizing unknown data, leading to poor performance in clinical scenarios that were not part of the training dataset [10,11]. Wang et al. evaluated the quality of DLS using computed tomography (CT) images taken each year and reported a substantial decline in performance over time [12]. Their results indicate that DLS cannot keep pace with changes in clinical practice as it evolves over time. In clinical practice, changes occur daily due to new technologies and improvements in accuracy, such as improvements in slice thickness and image quality of CT images, and the emergence of new surgical techniques. To keep up with these changes, DLS also needs frequent updates. This updating is essential for commercial DLS used in clinical settings.

The update approaches that vendors can implement include training DL models using a large dataset, incorporating local cases into the dataset, retraining the DLS model, and optimizing the hyperparameters of the model [10,11,13]. However, these solutions complicate the interpretation of the modified results. Accordingly, a working group recently recommended a qualitative or quantitative reassessment of contour quality [14]. This underscores the crucial role of contours in treatment planning, as they are essential for dose distribution assessment and optimization. As a result, post-update quality assessment is a necessary step. This is even more critical for commercial DLSs, as users typically have limited knowledge about how vendors modify these systems. Although several commercial DLSs are available, no studies have reported their post-update quality assessment because guidelines have only been reported recently [14]. In addition, commercial DLSs can delineate several dozen organs, and evaluating all of them takes time. Therefore, an efficient evaluation method is needed to minimize the burden on clinical workflows. However, the current guidelines do not provide detailed methods for assessing post-update DLS performance.

This study aimed to verify whether AI-Rad Companion Organs RT (AIRC; Siemens Healthineers, Erlangen, Germany), a commercial DLS, maintained the contour geometric accuracy post-update and to propose a streamlined validation method that minimizes the burden on clinical workflows.

## Materials and methods

Patient and image data

The study protocol was approved by the Institutional Review Board of St. Luke’s International Hospital (22-R118). All 109 participants were treated at our institution between 2021 and 2023. The patients underwent non-contrast CT of the head and neck, chest, abdomen, or pelvic region (i.e., head and back, n = 28; chest, n = 29; abdomen, n = 27; and pelvic region, n = 25). Patients undergoing head and neck CT scans were immobilized using a head and neck thermoplastic mask. Patients undergoing pelvic CT scans were instructed to evacuate using New Lecicarbon® suppositories (Zeria Pharmaceutical, Tokyo, Japan), urinate approximately 1 h before the scan, and drink 500 mL of water. An absorbable polyethylene glycol hydrogel spacer (SpaceOAR, Boston Scientific Corporation, Marlborough, USA) was used to reduce rectal dose in 15 patients with prostate cancer [15,16]. Planning CT images were acquired using a SOMATOM Confidence RT Pro (Siemens Healthineers, Erlangen, Germany) with a 120 kVp setting, 2.0 mm slice thickness, and either filtered back projection or DirectDensity reconstruction. The CT images of patients with implanted gold markers were processed to reduce metal artifacts. Organ motion, such as that of the lungs and heart, was not considered, and imaging was performed under free breathing.

The contours of the key organs, which served as the ground truth, were delineated by six experienced and qualified radiation oncologists following the Radiation Therapy Oncology Group atlas definitions and institutional guidelines. The contours delineated for each area were as follows:

Head and neck: Brain, brainstem, eye globe (right and left), lens (right and left), optic nerve (right and left), parotid gland (right and left), submandibular gland (right and left), and mandible.

Chest: Heart, lung (right and left), spinal cord, esophagus, and female breast (right and left).

Abdomen: Liver, spleen, and kidney (right and left).

Pelvis: Prostate, seminal vesicles, bladder, and rectum.

If inserted, the SpaceOAR was also defined. Previously, we evaluated the AIRC VA30 contours in the CT images in the head and neck region of 28 patients, chest of 29, abdomen of 27, and pelvic region of 25 [17]. For the comparison of post-update contours, VA50 and VA60 were used to delineate these organs in the same set of CT images. The superior and inferior borders of the rectal contours vary among radiation oncologists, introducing a potential bias in the evaluation. Therefore, as a pretreatment step, the rectal contour was limited to 1 cm above and below the prostate.

Evaluating contour quality across versions

All contours and CT images were transferred to RayStation (RaySearch Laboratories, Stockholm, Sweden) for analysis. The Dice similarity coefficient (DSC), maximum Hausdorff distance (HD), and mean distance to agreement (MDTA) were computed initially to identify contours with notable delineation differences between VA30 and VA50. These indices were calculated for comparison between VA30 and VA50. DSC values >0.9, MDTA <0.2 cm, and HD <1.0 cm are typical criteria for considering contours or regions as nearly identical, depending on the anatomical structure and clinical relevance [18-20]. However, this study applied stricter identification criteria: DSC of 0.99, MDTA of 0.05 cm, and HD of 0.2 cm. To evaluate the validity of these criteria, the set and standard criteria were defined as criteria A and B, respectively. If any single geometric indicator was below the criteria, it was judged to be a contour that was notably modified by the update. The same method was employed to evaluate VA50 and VA60.

To assess the identified contours, the ground truth was defined as the contours delineated by a radiation oncologist. The geometric indices for VA30, VA50, and VA60 were recalculated using a RayStation script (Appendix).

Statistical analysis

The geometric indices were classified according to the AIRC version (VA30, VA50, and VA60). Statistical analyses were performed in a Jupyter Notebook (https://jupyter.org/) using Python 3.11.4. Differences in the geometric indices across the AIRC versions were determined using the Wilcoxon signed-rank test, with significance set at *p* < 0.05.

Qualitative evaluation

To qualitatively assess the contours modified by the update, contours showing notable delineation differences were compared across different versions. In addition, their organs were qualitatively evaluated by comparing them to the ground truth.

## Results

Evaluating contour quality across versions

**Figure 1:**
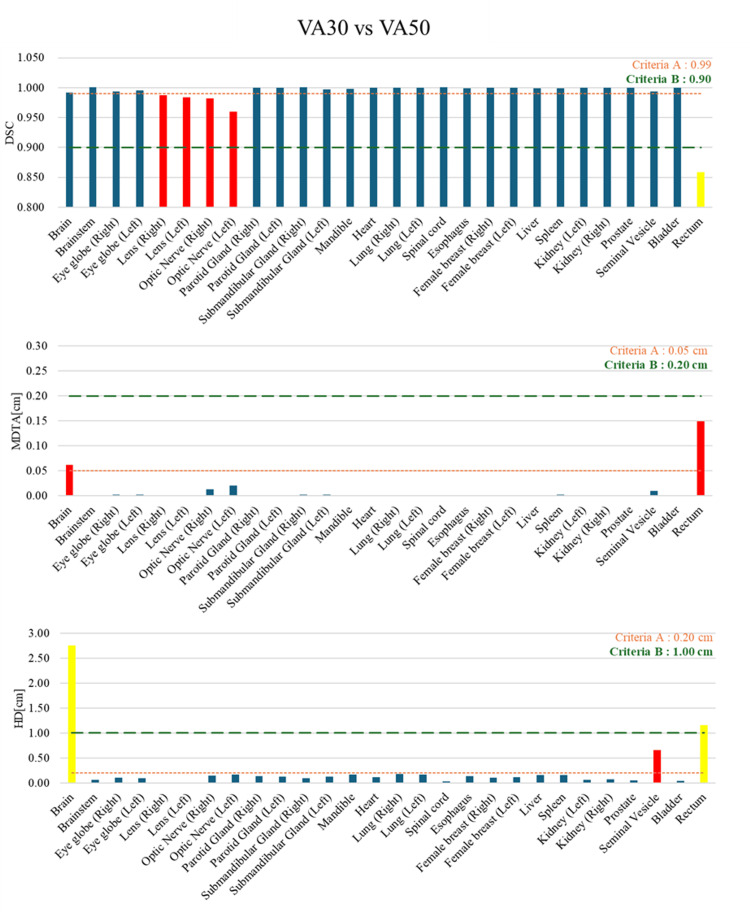
Bar graphs of the DSC, MDTA, and HD per contour derived from AIRC VA30 versus VA50 as the ground truth. The contours that did not meet criteria A and B are presented as red and yellow bars, respectively. The orange and green dot lines indicate criteria A and B, respectively. DSC, Dice similarity coefficient; HD, Hausdorff distance; MDTA, mean distance to agreement; AIRC, AI-Rad Companion Organs RT (Siemens Healthineers, Erlangen, Germany)

Figure 1 demonstrates bar graphs of the DSC, MDTA, and HD values per contour, derived from AIRC VA30 and VA50. The contours that did not meet even one of the criteria A were the brain, lens (right), lens (left), optic nerve (right), optic nerve (left), seminal vesicle, and rectum. In contrast, under criteria B, the brain and rectum contours did not meet the criteria. The mean DSCs of the brain and seminal vesicle were >0.99, while their mean HDs were 2.75 and 0.66, respectively. In contrast, the mean DSCs of the lens (right and left) and optic nerves (right and left) were <0.99; however, their MDTA and HD values were within the acceptable criteria. The mean HD of the rectum was 1.12 cm.

**Figure 2:**
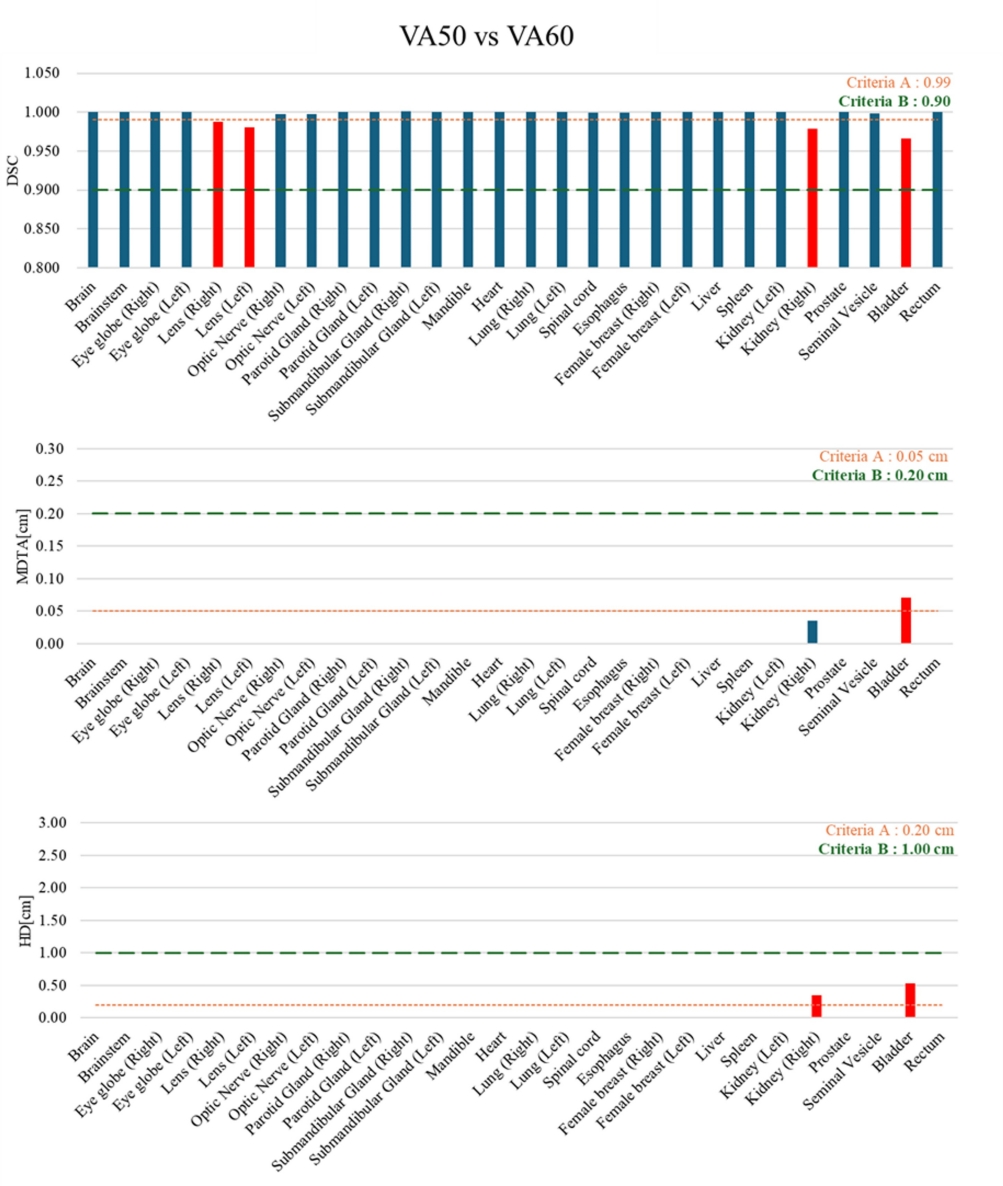
Bar graphs of the DSC, MDTA, and HD per contour derived from AIRC VA50 versus VA60 as the ground truth. The contours that did not meet criteria A and B are presented as red and yellow bars, respectively. DSC, Dice similarity coefficient; HD, Hausdorff distance; MDTA, mean distance to agreement; AIRC, AI-Rad Companion Organs RT (Siemens Healthineers, Erlangen, Germany)

Figure 2 demonstrates the bar graphs of the DSC, MDTA, and HD per contour derived from AIRC VA50 and VA60. The contours that did not meet even one of the criteria A were lens (right), lens (left), kidney (right), and bladder. In contrast, under criteria B, the contours of all organs met the criteria. The contours of the lens (right and left) exhibited the same trend as in Figure 1, with only the DSC exceeding the criteria. The bladder contour exceeded the criteria in all geometric indices, and only the MDTA of the kidney (right) contour was below the criteria.

Table 1 demonstrates the geometric indices of VA30, VA50, and VA60 calculated using the oncologist’s contour as the ground truth. When comparing VA30 and VA50, substantial differences were found for the brain in all geometric indices and the rectum in HD (p < 0.05). When comparing VA50 and VA60, substantial differences were found for the bladder in all geometric indices (p < 0.05). No considerable differences were observed in the other organs. The mean (range) pre-update and post-update DSCs for the brain were 0.94 (0.31-0.98) and 0.95 (0.33-0.99), and those for the bladder were 0.94 (0.90-0.96) and 0.96 (0.91-0.97), respectively. In addition, the mean (range) pre-update and post-update HDs for the brain were 2.94 (0.31-0.98) and 1.37 (0.33-0.99), and those for the bladder were 0.70 (0.35-1.00) and 0.54 (0.29-1.00) mm, respectively. The brain and bladder were more consistent with the ground truth post-update than pre-update. However, the mean (range) pre-update and post-update HDs for the rectum were 0.76 (0.40-1.16) and 1.13 (0.24-5.68) mm, respectively.

**Table 1:** Summary of averages and ranges for DSC, MDTA, and HD of VA30, VA50 and VA60 with radiation oncologist contour as ground truth. The geometric indices with significant differences in statistical analysis are bolded (*p*<0.05). DSC, Dice similarity coefficient; HD, Hausdorff distance; MDTA, mean distance to agreement

	DSC				MDTA				HD				
	VA30	VA50		VA60	VA30	VA50		VA60	VA30	VA50			VA60
Brain	0.94 (0.31–0.98)	0.95 (0.33–0.99)		–	0.16 (0.09–0.77)	0.10 (0.05–0.73)		–	2.94 (1.99–4.69)	1.37 (0.74–4.69)			–
	p = 6.15 × 10^−4^		–		p = 5.83 × 10^−8^		–		p = 2.33 × 10^−9^		–		
Lens (right)	0.65 (0.34–0.81)	0.65 (0.34–0.81)		0.65 (0.34–0.81)	0.05 (0.02–0.13)	0.05 (0.02–0.13)		0.05 (0.02–0.13)	0.21 (0.10–0.50)	0.21 (0.10–0.50)			0.21 (0.10–0.50)
	p = 0.934		p = 1.000		p = 0.985		p = 1.000		p = 1.000		p = 1.000		
Lens (left)	0.63 (0.06–0.80)	0.63 (0.07–0.80)		0.63 (0.07–0.80)	0.05 (0.00–0.12)	0.05 (0.00–0.12)		0.05 (0.00–0.12)	0.18 (0.00–0.40)	0.18 (0.00–0.40)			0.18 (0.00–0.40)
	p = 0.855		p = 1.000		p = 0.985		p = 1.000		p = 1.000		p = 1.000		
Optic nerve (right)	0.43 (0.18–0.66)	0.43 (0.18–0.64)		–	0.24 (0.14–0.41)	0.24 (0.14–0.41)		–	0.87 (0.39–1.73)	0.92 (0.48–1.71)			–
	p = 0.883		–		p = 0.806		–		p = 0.363		–		
Optic nerve (left)	0.43 (0.00–0.77)	0.42 (0.11–0.84)		–	0.20 (0.06–0.58)	0.22 (0.05–0.51)		–	0.72 (0.17–1.82)	0.84 (0.17–2.26)			–
	p = 0.629		–		p = 0.629		–		p = 0.583		–		
Seminal vesicle	0.63 (0.36–0.86)	0.63 (0.36–0.86)		–	0.24 (0.08–0.62)	0.24 (0.09–0.61)		–	1.33 (0.41–2.98)	1.60 (0.58–3.00)			–
	p = 0.846		–		p = 0.628		–		p = 0.225		–		
Rectum	0.85 (0.74–0.92)	0.85 (0.68–0.94)		–	0.15 (0.07–0.23)	0.14 (0.04–0.33)		–	0.76 (0.40–1.16)	1.13 (0.24–5.68)			–
	p = 0.808		–		p = 0.705		–		p = 0.0411		–		
Kidney (right)	–	0.92 (0.83–0.95)		0.92 (0.85–0.95)	–	0.12 (0.07–0.30)		0.11 (0.07–0.27)	–	0.88 (0.41–1.57)			0.86 (0.47–1.49)
	–		p = 0.938		–		p = 0.924		–		p = 0.586		
Bladder	–	0.94 (0.90–0.96)		0.96 (0.91–0.97)	–	0.12 (0.08–0.23)		0.08 (0.06–0.16)	–	0.70 (0.35–1.00)			0.54 (0.29–1.00)
	–		p = 2.66 × 10^−5^		–		p = 8.72 × 10^−7^		–			p = 0.010	

Qualitative evaluation

The lens and optic nerve contours in VA50 were only extended by one slice (0.2 cm) in the superior or inferior direction compared with those in VA30, though the contoured areas in VA30 were perfectly aligned. The seminal vesicle in VA50 was extended by 1-10 slices in the superior direction compared with that in VA30. The intestinal and lymphatic vessels near the seminal vesicles were included in the contours. However, the seminal vesicle was perfectly aligned in the contoured areas of VA30. The kidney (right) contour in VA60 did not match the contour outline in VA50; however, there was nearly no difference.

**Figure 3:**
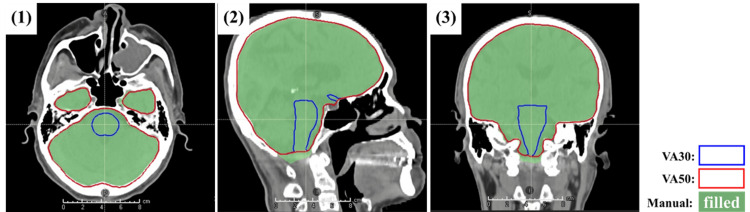
Brain contours delineated with different AIRC versions and the ground truth of brain contours determined by an oncologist. Numbers 1, 2, and 3 indicate transverse, sagittal, and coronal sections, respectively. AIRC, AI-Rad Companion Organs RT (Siemens Healthineers, Erlangen, Germany)

Figure 3 shows the brain contours delineated with different AIRC versions and their ground truth. The case presented in the figure referred to that of a patient with median brain DSC in VA50. The brain contour in VA30 covers the entire brain, while the brain contour in VA50 excludes the brainstem region. No differences were noted in the areas of the brain, excluding the overlapping regions.

**Figure 4:**
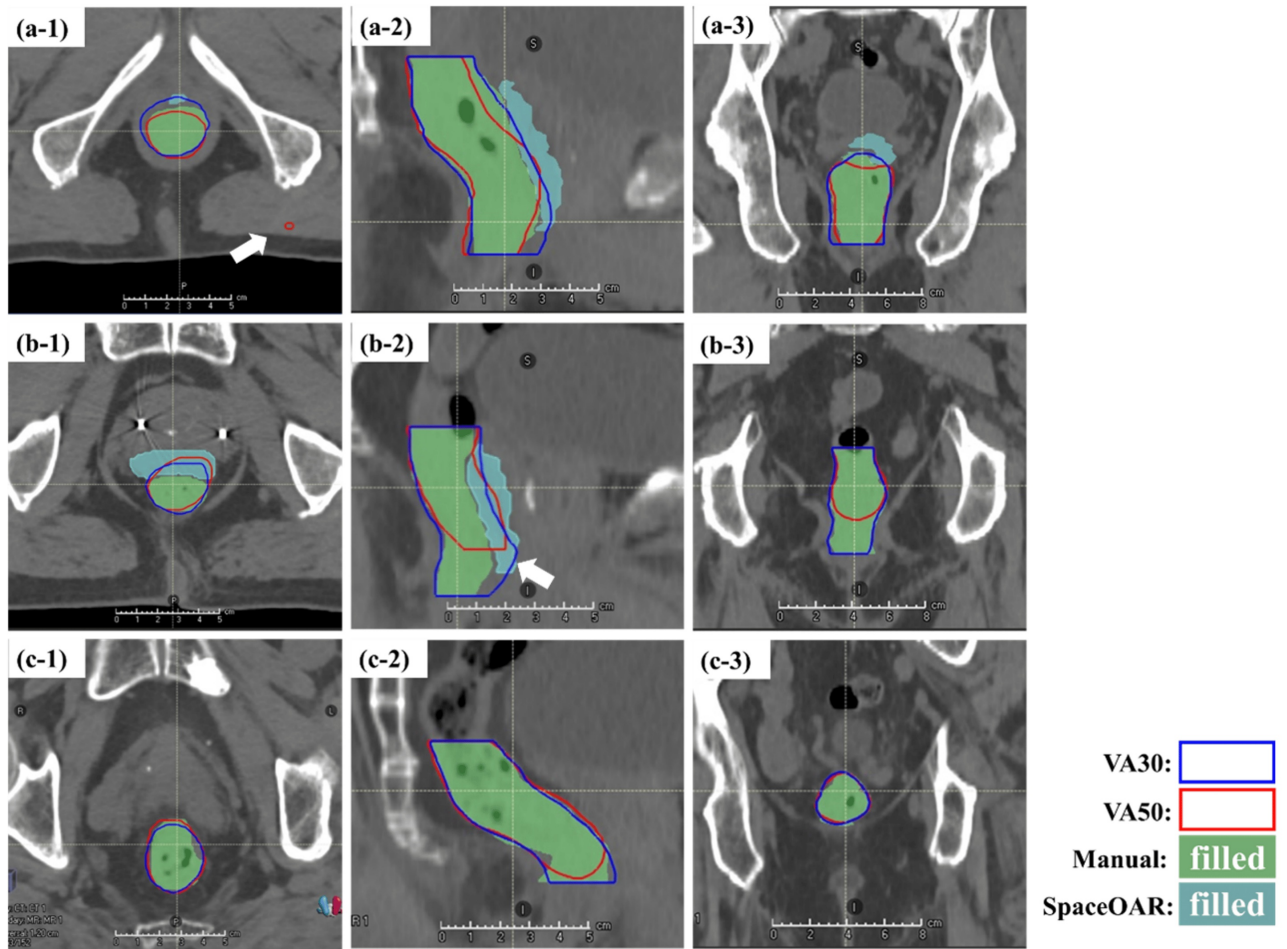
Rectum contours delineated with different AIRC versions, ground truth of rectum contours determined by an oncologist, and SpaceOAR. (a) Maximum HD for the rectum. (b) Minimum DSC for the rectum. (c) Median DSC for the rectum. Numbers 1, 2, and 3 indicate transverse, sagittal, and coronal sections, respectively. DSC, Dice similarity coefficient; HD, Hausdorff distance, AIRC, AI-Rad Companion Organs RT (Siemens Healthineers, Erlangen, Germany); SpaceOAR by Boston Scientific Corporation, Marlborough, USA

Figure 4 shows the rectum contours delineated with different AIRC versions and their ground truth. The cases presented in the figure were those of patients with maximum HD (Figure 4a), minimum DSC (Figure 4b), and median DSC (Figure 4c) for the rectum in VA50. In the case of maximum HD, an isolated region of interest (ROI) in an area was clearly different from the rectum. In the case of the minimum DSC, the VA50-delineated rectal contours were shorter in the inferior direction than the VA30-delineated contours. In cases without SpaceOAR, a short rectum of VA50 was similarly depicted. The outline of the rectum between VA30 and VA50 did not match.

**Figure 5:**
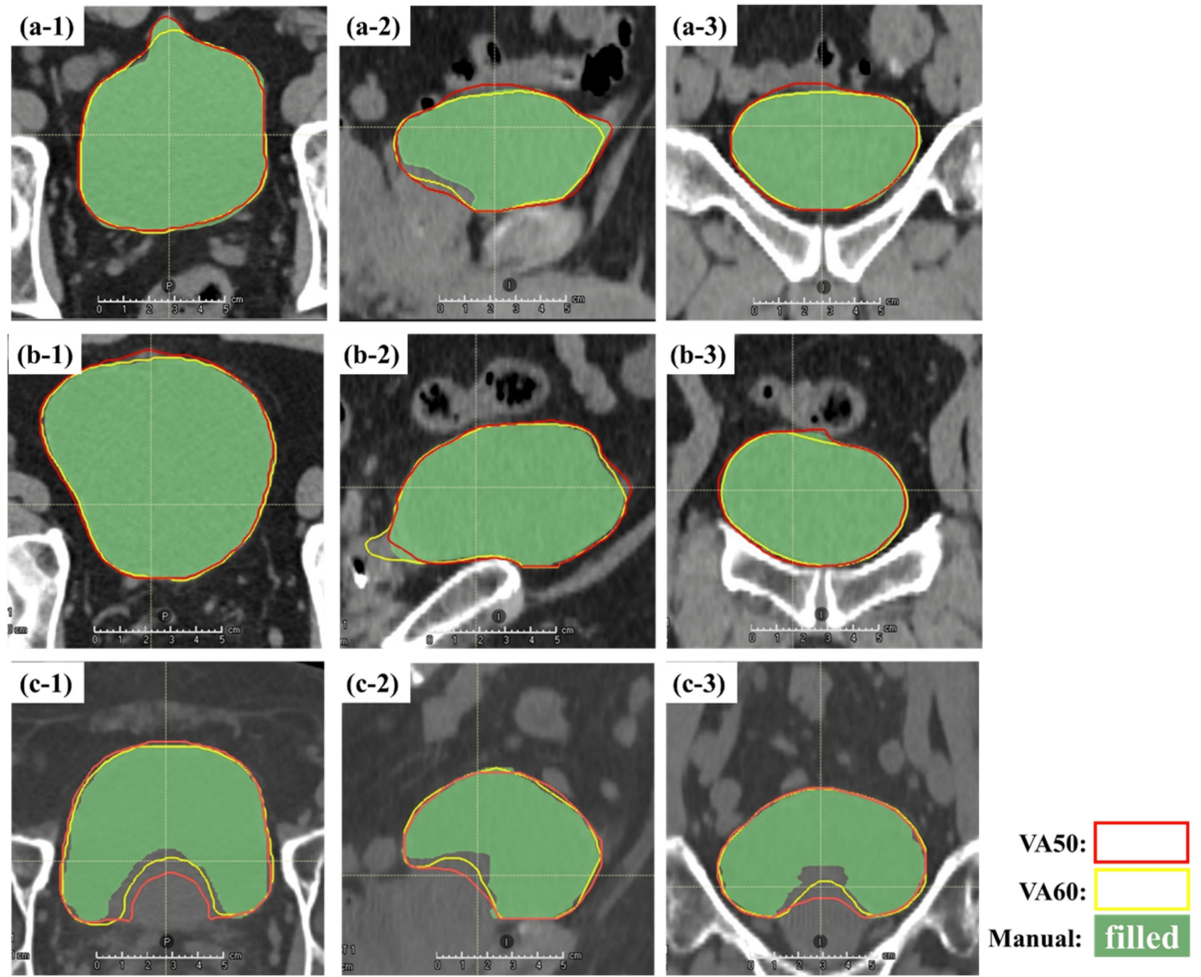
Bladder contours delineated with different AIRC versions and ground truth of bladder contours determined by an oncologist. (a) Median DSC for the bladder. (b) Maximum HD for the bladder. (c) Median DTA for the bladder. Numbers 1, 2, and 3 indicate transverse, sagittal, and coronal sections, respectively. DSC, Dice similarity coefficient; HD, Hausdorff distance, AIRC, AI-Rad Companion Organs RT (Siemens Healthineers, Erlangen, Germany); DTA, Distance to agreement

Figure 5 shows the bladder contours delineated with different AIRC versions and the ground truth of bladder contours. The bladder contour in VA50 included the intestinal part adjacent to the bladder but was excluded in VA60. However, the irregularly protruding bladder was accurately depicted in VA50 but not in VA60 (Figure 5a). In most cases, the bladder contour improved at the border with the prostate (Figure 5a and Figure 5c). In one case, the VA60 bladder incorrectly included the prostate (Figure 5b). The outline of the bladder between VA50 and VA60 did not match.

## Discussion

The detection of contouring errors cannot be ascertained solely through geometric indicators; thus, a qualitative assessment is essential. However, reviewing all contours after a DLS version update is not practical. Therefore, it is necessary to limit the contours evaluated according to specific criteria. In this study, the number of contours evaluated was limited by establishing criteria for geometric indicators. We believe that the minimum required verification can be achieved using the method proposed in this study, even when the DLS at each facility is updated. Among the 28 contours evaluated, the contours of nine organs did not meet criteria A. Statistical analysis revealed that the brain, rectum, and bladder contours differed substantially across the AIRC version. In particular, the pre-update rectum contour had a mean (range) HD of 0.76 (0.40-1.16); however, the post-update rectum exhibited a lower-quality mean (range) HD of 1.13 (0.24-5.68). This information is not disclosed in the specifications or other documentation provided by the vendor. This finding highlights the need for post-update quality assessment.

The contours of the lens and optic nerve did not meet the DSC criteria; however, no substantial differences were observed across the evaluated versions. A slight difference was noted in the qualitative evaluation. This implies that not all contours that did not meet the DSC criteria differed between VA50 and VA30. Notably, DSC can become too sensitive in the evaluation of small contours, leading to a disproportionately high sensitivity to minor discrepancies in contour alignment [14]. Therefore, to identify contours with notable delineation differences, a contour volume-independent index, such as MDTA or HD, must be used.

The contours of the seminal vesicles did not meet the HD criteria, and the kidney (right) contours also failed to meet the DSC and HD criteria. These two organs underwent different modifications. The seminal vesicles perfectly matched only in the VA30 region. Thus, the seminal vesicles may not have been modified by the DLS model. There are two possible modifications in the processing. The AIRC delineates contours through a two-step process. The first step utilizes multi-scale deep reinforcement learning to extract target regions from input images [21]. The second step involves contour delineation based on the extracted image [22]. If the first step was modified, most contours before and after the update would remain identical, suggesting that changes in the seminal vesicles were due to this alteration. The other possible modification involves adjustments to the delineated contours themselves, such as cropping part of a contour based on the boundaries of adjacent organs. These processes are likely responsible for the observed changes in the contours of the seminal vesicles. Conversely, the kidney (right) contours in VA30 and VA50 did not match, suggesting that the DLS model was also modified. However, if only the contour handling was updated, as with the seminal vesicles, the changes were easy to identify. When the DLS model itself is updated, as with the kidney (right), evaluation becomes more complicated. If the DLS model was modified and the contours differed significantly from the previous version, users should evaluate them quantitatively against the ground truth, which consists of contours delineated by radiation oncologists. This assessment is essential, as it helps determine whether the modifications have improved or degraded the contour quality, even if the specific changes are unclear.

The better values of all geometric indices in VA50 indicate improvements in the contour quality of the brain. The images of patients with a median DSC indicated that this result was due to the modified treatment of the overlapping areas between the brain and brainstem (Figure 3). No differences were noted in the areas of the brain excluding the overlapping regions. Therefore, this change does not reflect an update to the DLS model itself but rather an adjustment in the approach to contour handling. This modification influences the optimization and evaluation of the treatment plan. Thus, users should be aware of this modification before using AIRC VA50. In addition, modifications to treatment planning protocols should be taken into account.

The rectum did not meet the criteria for any geometric indices, and substantial differences in HD were observed between the models. The maximum HD was 5.67 cm, indicating that the DLS model depicting the rectal contour was modified. Furthermore, rectal images with the maximum HD revealed that VA50 delineated isolated irregular contours that were not found in VA30 (Figure 4a). These irregularities suggest errors in the modified DLS model. This was attributed to unidentifiable factors present in the DLS model. Notably, these isolated contours occurred in only one out of the 25 patients. After modifying the DLS model, it may be necessary to obtain a sample of patient data comparable with those used in this study to ensure that the contours are accurate and error-free.

In the case of the minimum DSC, the VA50-delineated rectal contours were shorter than the VA30-delineated contours (Figure 4b and Figure 4c). As confirmed throughout the case series, the rectal contour in VA50 was short in nine of 25 patients. Portions 1 cm above and below the prostate contour were deleted to focus on the rectal contour within the irradiated area. However, the rectal contour in this area was typically larger than the selected size. According to the European Society for Radiotherapy and Oncology Advisory Committee on Radiation Oncology Practice consensus guidelines, rectal contouring should end at approximately 2 cm below the lowest apical prostate contour [23]. Consequently, rectal contours should be checked during treatment planning and may require extension in the caudal direction depending on each case. In summary, our results indicate that modifications to the DLS model in AIRC can result in incorrect rectal contours. In radiotherapy, it has been demonstrated that variations in contouring can lead to variations in dose distribution [24,25]. Kawula et al. evaluated the impact of DLS-delineated rectum, bladder, and prostate contours on dose optimization in radiotherapy for prostate cancer patients [26]. They reported that although all contours achieved a mean DSC of 0.85 or higher, the worst case exhibited a low Gamma Pass rate (3 mm/3%) of 71%. In the present study, the mean DSC for rectal contours comparing VA30 and VA50 was 0.87 (Figure 1). These findings suggest that updates to the DLS may influence dose optimization.

The images of bladder contours indicated that the DLS model depicting bladder contours was modified (Figure 5). The better values provided by VA60 for all geometric indices indicate improvements in the bladder contour’s quality. Although the bladder contour in VA60 was improved and excluded the bowel, it did not accurately capture the irregularly protruding parts of the bladder. While a correlation between geometric metrics and qualitative evaluation has been demonstrated, it does not guarantee clinical acceptability [20]. Thus, despite improvements in the geometric indices, final human confirmation remains essential in clinical use.

In this study, the proposed method involves calculating geometric indices based on the contours obtained before and after updates rather than using physician-delineated contours as the ground truth from the outset. This approach allows for the direct evaluation of the effects of model updates on contour changes. If geometric indices were calculated from physician-delineated contours as the ground truth and then compared across different versions, false negatives could occur. In other words, changes in the contours may not reflect changes in the geometric indices, which is an undesirable outcome when verifying contour modifications. Moreover, when using physician-delineated contours as the ground truth, geometric indices vary across organs; thus, establishing a threshold for significant differences is challenging. Given these limitations, geometric indices may be more appropriately calculated based on pre- and post-update contours, and a fixed criterion for evaluating DLS updates must be established.

When applying the proposed method using criteria B, only the brain and rectum were identified as organs with notable differences. The evaluation revealed that modifications to the brain and rectum contours have significant clinical effects and should be assessed accordingly. However, the kidney (right) and bladder, for which the DLS model used was modified, also require assessment, as the implications of these modifications on the generated contours remain uncertain. Therefore, criteria B may not be appropriate. Although criteria A typically focuses on smaller organs, it offers a safer approach for evaluation.

Limitation

First, the contours of six radiation oncologists were used as the ground truth. Although they followed the institution’s guidelines, variations in contouring may have occurred within and among raters. This may have introduced bias in the geometric indices presented in this study. Second, 15 patients underwent SpaceOAR insertion in the pelvic region. A study reported that surgical interventions such as SpaceOAR insertion can significantly reduce DLS quality [10]. Thus, when updating the DLS model, particularly for the rectum, the degradation caused by SpaceOAR may either improve or worsen contour accuracy. These effects may need to be stratified and verified based on the presence or absence of SpaceOAR. Third, this study used only a single DLS software, AIRC. As a result, our findings may not be generalizable to other commercial DLS systems. However, it is essential that other DLSs also be evaluated for contour accuracy following updates. Finally, while this study demonstrated that updates to the DLS can lead to contouring errors, we did not assess whether these errors resulted in significant changes in dose distribution or treatment outcomes. Future research should investigate the clinical impact of contour quality degradation to determine its potential consequences for radiotherapy planning and patient outcomes.

## Conclusions

In this study, the contour qualities of AIRC VA30, VA50, and VA60 were assessed, focusing on contours altered by the update. The update to the AIRC version resulted in changes to the approach for brain contour handling and modifications to the DLS model for the rectum and bladder. Notably, the quality of rectal contours after the update was found to be inferior to that before the update. The findings of this study highlight the need for reassessing the contour quality for AIRC and other regularly updated commercial DLS models. The results indicate that the proposed method can reduce the burden on clinical workflows while appropriately evaluating post-update DLS.

## Appendices

**Table 2:** Example script code for calculating geometric indices in RayStation This code searches for a specified Bodysite in the database and automatically computes geometric indices. Therefore, a target Bodysite must be specified for validation. RayStation by RaySearch Laboratories, Stockholm, Sweden

1	#Example script code for calculating geometric indices in Raystation
2	from connect import *
3	import csv
4	
5	Bodysite = ['AIRC_Abd']
6	db = get_current('PatientDB')
7	
8	error_log = []
9	#Create a list of ROI names to compare
10	RoiList_post = ["Liver_VA70","Spleen_VA70","Kidney (Left)_VA70","Kidney (Right)_VA70"]
11	RoiList_pre = ["Liver_VA50","Spleen_VA50","Kidney (Left)_VA50","Kidney (Right)_VA50"]
12	
13	NumOfRois = len(RoiList_pre)
14	
15	#File name
16	#folder_path = 'C:/your/folder/path'
17	folder_path = 'E:/AIRC_Study/ROI_Similarity_Va40Va50/abd'
18	filename = 'ContourEval_Abd.csv'
19	path = '{0}/{1}'.format(folder_path,filename)
20	
21	with open(path, 'w') as f:
22	writer = csv.writer(f)
23	writer.writerow(["PatientID", "Roi Name", "DICE", "MeanDTA", "HausdorffDistance"])
24	
25	for p_id in Bodysite:
26	patient_info = db.QueryPatientInfo(Filter={'BodySite':p_id})
27	print(len(patient_info))
28	for i in range(len(patient_info)):
29	patient = db.LoadPatient(PatientInfo = patient_info[i])
30	#find a case with saved ROIs before and after update.
31	for x in range(100):
32	
33	case = patient.Cases[x]
34	case.SetCurrent()
35	
36	if case.BodySite == p_id:
37	break
38	
39	examination = get_current("Examination")
40	case_name = case.CaseName #case name
41	ex_name = examination.Name #CT name
42	
43	for i in range(NumOfRois):
44	#try to calculate geometric indices for each ROI.
45	try:
46	if patient.Cases[case_name].PatientModel.StructureSets[ex_name].RoiGeometries[RoiList_pre[i]].HasContours() and patient.Cases[case_name].PatientModel.StructureSets[ex_name].RoiGeometries[RoiList_post[i]].HasContours():
47	print ('Comparison between', RoiList_post[i], ' and ' ,RoiList_pre[i])
48	CompareData = patient.Cases[case_name].PatientModel.StructureSets[ex_name].ComparisonOfRoiGeometries(RoiA = RoiList_post[i],
49	RoiB = RoiList_pre[i],
50	ComputeDistanceToAgreementMeasures = True)
51	dice = CompareData["DiceSimilarityCoefficient"]
52	meanDTA = CompareData["MeanDistanceToAgreement"]
53	HausdorffDistance = CompareData["MaxDistanceToAgreement"]
54	
55	#File name
56	with open(path, 'a') as f:
57	writer = csv.writer(f)
58	writer.writerow([str(patient.PatientID).zfill(10), RoiList_post[i], dice, meanDTA, HausdorffDistance])
59	
60	except Exception as e:
61	error_log.append([patient.PatientID, RoiList_post[i], RoiList_pre[i]])
62	
63	#If an error occured, output the ID of the patinet.
64	if error_log:
65	print("\n Error found:")
66	for dsc, mdta, hd in error_log:
67	print("patientID: {0}, comparing {1} and {2}".format(dsc, mdta, hd))
68	else:
69	print("\n Error not found")
